# Ureter Regeneration–The Proper Scaffold Has to Be Defined

**DOI:** 10.1371/journal.pone.0106023

**Published:** 2014-08-27

**Authors:** Tomasz Kloskowski, Arkadiusz Jundziłł, Tomasz Kowalczyk, Maciej Nowacki, Magdalena Bodnar, Andrzej Marszałek, Marta Pokrywczyńska, Małgorzata Frontczak-Baniewicz, Tomasz A. Kowalewski, Piotr Chłosta, Tomasz Drewa

**Affiliations:** 1 Chair of Regenerative Medicine, Department of Tissue Engineering, Collegium Medicum, Nicolaus Copernicus University, Bydgoszcz, Poland; 2 Institute of Fundamental Technological Research, Polish Academy of Sciences, Warsaw, Poland; 3 Department of Clinical Pathomorphology, Collegium Medicum, Nicolaus Copernicus University, Bydgoszcz, Poland; 4 Electron Microscopy Platform, Mossakowski Medical Research Centre, Polish Academy of Sciences, Warsaw, Poland; 5 Urology Department, Jagiellonian University, Krakow, Poland; 6 Urology Department, Nicolaus Copernicus Hospital, Toruń, Poland; Universidade de Sao Paulo, Brazil

## Abstract

The aim of this study was to compare two different acellular scaffolds: natural and synthetic, for urinary conduit construction and ureter segment reconstruction. Acellular aortic arch (AAM) and poly(L-lactide-*co*-caprolactone) (PLCL) were used in 24 rats for ureter reconstruction in both tested groups. Follow-up period was 4 weeks. Intravenous pyelography, histological and immunohistochemical analysis were performed. All animals survived surgical procedures. Patent uretero-conduit junction was observed only in one case using PLCL. In case of ureter segment reconstruction ureters were patent in one case using AAM and in four cases using PLCL scaffolds. Regeneration of urothelium layer and focal regeneration of smooth muscle layer was observed on both tested scaffolds. Obtained results indicates that synthetic acellular PLCL scaffolds showed better properties for ureter reconstruction than naturally derived acellular aortic arch.

## Introduction

The main indication for ureter reconstruction are iatrogenic injuries, muscle invasive ureter cancer is rare [1], [2]. The gold standard for urinary diversion following radical cystectomy and ureter segment reconstruction is use of the ileal conduit [3], [4]. Use of tissue engineering techniques for urinary conduit creation and ureter segment reconstruction can eliminate most of complications related to currently used procedures, and when combined with laparoscopic approach can reduce time of surgery by 1.5–2 h and increase patient survival rate [5].

Ideal material for ureteral conduit creation and ureter segment reconstruction should be easily accessible, impermeable for urine, non-immunogenic, guarantee future remodeling, and should possess appropriate conditions for cell growth and migration [6], [7]. Many reports showed that scaffold preseeding with autologous stem cells derived from adipose tissue or bone marrow enhance vascularization and regeneration of reconstructed tissues, but acellular scaffolds have a possibility to be applied into the clinic [8], [9]. Constructs of appropriate scaffold that will regenerate structure of ureter by itself will be ideal solution to eliminate necessity of autologous tissue biopsy. Such approach will be less harmful for patients and will shorten procedure by elimination of time necessary for cell culturing. Acellular scaffolds can be promising as a potential “of-the-shelf” product.

Till now we have no clear answer what kind of material is the best for ureter reconstruction. If such material will be discovered it will have a great impact on medical ureteral reconstruction procedures. The aim of the present study was to compare two types of scaffolds for ureter reconstruction: naturally derived rat acellular aortic arch (AAM) and electrospun nanofibrous scaffold made of synthetic poly(L-lactide-*co*-caprolactone) (PLCL).

Naturally derived vascular grafts are composed of proteins, glycosaminoglycans, different collagen types (I, III, IV, VI, VIII, XV, XVIII), laminins, elastin, fibrilin, proteoglycans, vWF, and other components which support cell adhesion, migration and proliferation [10]. Disadvantages are different structure from native tissue and cytotoxic induction after decellularization procedure, and porous structure that can perfuse urine. These factors may cause inflammation and disorder of cell colonization [11], [12]. Decellularization process, since its first use, is now more precisely described and allows to obtain acellular scaffold not only from tissues but also from whole organs like kidney [13]–[15].

Electrospun nanofibers are created when liquid jet of viscoelastic fluid is subjected to a high electric field (0.1−1 kV/cm). In such conditions a filament of polymer solution or melt starts to make looping motion caused by a charge repulsion. This movement creates very thin fibers (from several nm to about 2 µm) of the polymer. These fibers form material of very special properties. They are very similar to Extracellular Collagen Matrix (fiber size 50–500 nm), they are treated by cells as their native environment. Nonwoven nanofibrous membrane is colonized by the cells thus they can form a “pseudo-tissue”. If nanofibrous membrane is created from biodegradable and biocompatible polymer (e.g. poly(L-Lactide-co-caprolactone)) the material has no negative impact on the cells – is gradually hydrolyzed by cells and replaced by a native collagen. Aliphatic polyesters are special group of polymers – they degrade in the environment of organism to create natural metabolites or their non-toxic analogues. Most known and used in surgery and tissue engineering are (co)polymers of lactic acid. The final product of degradation of poly(lactic acid) is lactic acid. Thick implants produce noticeable amounts of this acid (pKa = 3.83) that can irritate surrounding tissue. Nanofibrous implants have highly porous structure and contain far less material than materials made of solid poly(lactic acid). There is a family of polyesters – aliphatic polycarbonates that generate only very weak carbonic acid and therefore they have limited impact on the tissue irritation [16]–[18]. Polyesters are created *de novo*, no animal material has to be used to synthesize them. They can be combined with other compounds and it is possible to control their shape, size, porosity, cell attachment, or biodegradability [19]–[21]. Disadvantages are possibility of inflammation inducted by a too long degradation time for PLCL [22], [23].

Our experiment was divided into two stages. In the first stage, urinary conduit construction was performed to test proximal anastomosis of ureter with scaffold and to check continuity of urinary conduit lumen. In the second stage reconstruction of important clinically ureter segment defect was performed to test proximal and distal anastomosis of ureter with scaffold and to check passage of urine through reconstructed segment.

## Materials and Methods

### Scaffolds

Aortic arches were obtained from 12 donor Wistar rats. The decellularization process involved aortic arch incubation at 4°C in 0.2% Triton X-100 (Sigma, Germany) and 26.5 mmol/L ammonium hydroxide (Sigma, Germany) solution for 14 days. Aortic arches were then washed in deionized water for 3 days and disinfected by storage in phosphate buffered saline (PBS) with antibiotics (PAA, Austria) until implantation. Acellularity was confirmed by routine hematoxylin and eosin (HE) staining. Dimensions of implanted scaffolds was: length −1 cm, diameter –3–4 mm for PLCL and 2 mm for aortic arch.

Tubular nanofibrous scaffolds were produced using electrospinning method. The electrospinning process has been described in details elsewhere [24], [25]. In our experiment, the spinning distance was 20 cm, the electric potential was 15 kV, and the solution throughput was 0.500 µl/h. A rotating brass rod (1000 rpm, diameter of 4 mm) served as a nanofibrous mat collector. The polymer used for electrospinning was poly(L-lactide-*co*-caprolactone) (PLCL), which was composed of 70% L-lactide and 30% caprolactone units and is known commercially as Purasorb 7015 (Purac - Corbion, Gorinchem, Netherlands). The electrospinning solution was made of 9% polymer dissolved in a mixture of solvents (chloroform+dimethylformamide, mass proportions 16∶1). A scanning electron microscopy analysis was performed to visualize the biomaterial structure.

### PLCL degradation test

Biomaterials were implanted into the rat abdomen. Ten Male Wistar rats were anaesthetized with intraperitoneal sodium pentobarbitone in dose 50 mg/kg on body weight. Post*-*operative analgesia with opiate based pain killers was provided. All animals survived the surgical protocol and in all cases the implantation sites in surgical wounds healed without complications. Six weeks after PLCL scaffold implantation rats were scarified, implanted biomaterials were removed and prepared for electron microscopy evaluation.

The samples were prepared with ‘regular’ ice-cold fixative with 2% paraformaldehyde (Sigma, Germany) and 2.5% glutaraldehyde (Sigma, Germany) in 0.1 M sodium cacodylate buffer, pH 7.4 (Sigma, Germany), as was described earlier [26]. The specimens were handled with the typical ice-cold fixative (see above) post-fixed in 1% (w/v) OsO_4_ (Sigma, Germany) solution in deionized water, dehydrated in an ethanol gradient, and encased in epoxy resin (Epon 812, Sigma, Germany). Ultrathin (60 nm) sections were prepared as described earlier [26]. Specimens were examined in a transmission electron microscope (JEM-1200EX, Jeol, Japan).

### Cytotoxic assay

Both scaffolds were incubated in standard culture medium DMEM Ham’s F12 (PAA, Austria) with 10% FBS and antibiotics (Sigma, Germany) for 2 week in incubator (36°C, 5% CO_2_). Such obtained conditioned medium was next added to the rat smooth muscle cell line (CRL 2018, ATCC, USA) after 24 h incubation on 24-well plate. Cells were seeded with density 5×10^4^ on each well and cultured in standard culture medium. Cytotoxicity of scaffolds was measured using MTT assay after 24 48, and 72 h incubation with conditioned medium (in control with standard medium). Results were presented as mean from ten independent measurements.

### Cell growth analysis

Acellular aortic arch scaffold was attached do the well of 6-weel plate using needles, considering its small size. PLCL scaffold was placed in inserts (Cell Crown, Scaffdex, Finland) which were next placed in 24-well plate. Rat smooth muscle cell line (CRL2018, ATCC, USA) were seeded on each scaffold with density 5×10^5^ on 1.9 cm^2^. Cell growth on outer scaffold surface was performed using MTT assay (Sigma, Germany).

### Surgical procedure

This experiment was approved by the Committee on the Ethics of Animal Experiments of the University of Technology and Life Science in Bydgoszcz, Poland (no. 3/2012). Twenty-four Wistar rats (10 weeks old) were randomly divided into two equal groups (12 rats on each), each group was additionally divided into two equal subgroups (6 for aortic arch scaffold, 6 for nanofibrous tubular scaffold).

### Urinary conduit construction

The isolated right ureters were cut near the bladder. The distal end of the acellular aortic arch scaffold (Group 1A) or nanofibrous scaffold (Group 1B) scaffold was implanted into previously formed channel in the muscle layer and fixed to the fascia and skin. The ureters were anastomosed end-to-end to proximal end of the scaffold using 8-0 non-absorbable sutures. No stenting of the ureteral anastomosis or drainage was done.

### Ureter segment reconstruction

In the middle part of right ureter 1 cm defect was replaced with the acellular aortic arch scaffold (Group 2A) or nanofibrous scaffold (Group 2B) anastomosed with ureter stumps by end-to-end anastomosis using 8-0 non-absorbable sutures. Catheter (3 cm length, 0,6 mm diameter, GALMED, Bydgoszcz, Poland) was introduced into reconstructed ureter site on all follow-up period.

The follow up period was 4 weeks. The patency of the ureters and conduits was assessed by intravenous pyelography at 4 weeks using X-ray Actube Dental 5D2 with exposures at 60 kV and 6 mA.

### Changes in kidney size

Evaluation of kidneys size on the operated side compared to normal kidneys was performed on the basis of photographic documentation (ImageJ, NIH, USA). Kidneys size was measured by summing their length and width in 2D picture, results were presented in percentage.

### Histological and immunohistochemical analysis

The constructed specimens were fixed in 10% neutral buffered formalin and embedded in paraffin. Cross-sections of the whole reconstructed segments and kidneys were prepared. Histological analysis with HE staining was performed. The connective tissue components and muscle layer were stained according to Masson staining.

For confirmation of smooth muscle layer regeneration immunohistochemical analysis using anti-smooth muscle α-myosin heavy chain (α-SMM, Abcam, Great Brittan). This analysis was performed according to the procedure described previously [27]. Briefly, tissue sections were incubated with primary antibody against α-SMM (dilution 1∶400). After washing, the sections were overlaid with peroxidase-conjugated anti-mouse secondary antibody (EnVision/HRP anti Mouse; Dako, Denmark). Stained samples were analyzed using light microscopy by two independent pathologists.

### Statistical analysis

Cytotoxic differences between standard and conditioned medium was evaluated using t-Student or Cochran-Cox. The significance level p<0.05 was used as reliable.

In order to detect dependence between tested scaffolds and reconstruction result, Fisher’s exact test and two fraction test were used (Statistica 10.0, StatSoft, USA) because of small class number. The significance level p<0.05 was used as reliable.

## Results

### Scaffolds

**Figure 1:**
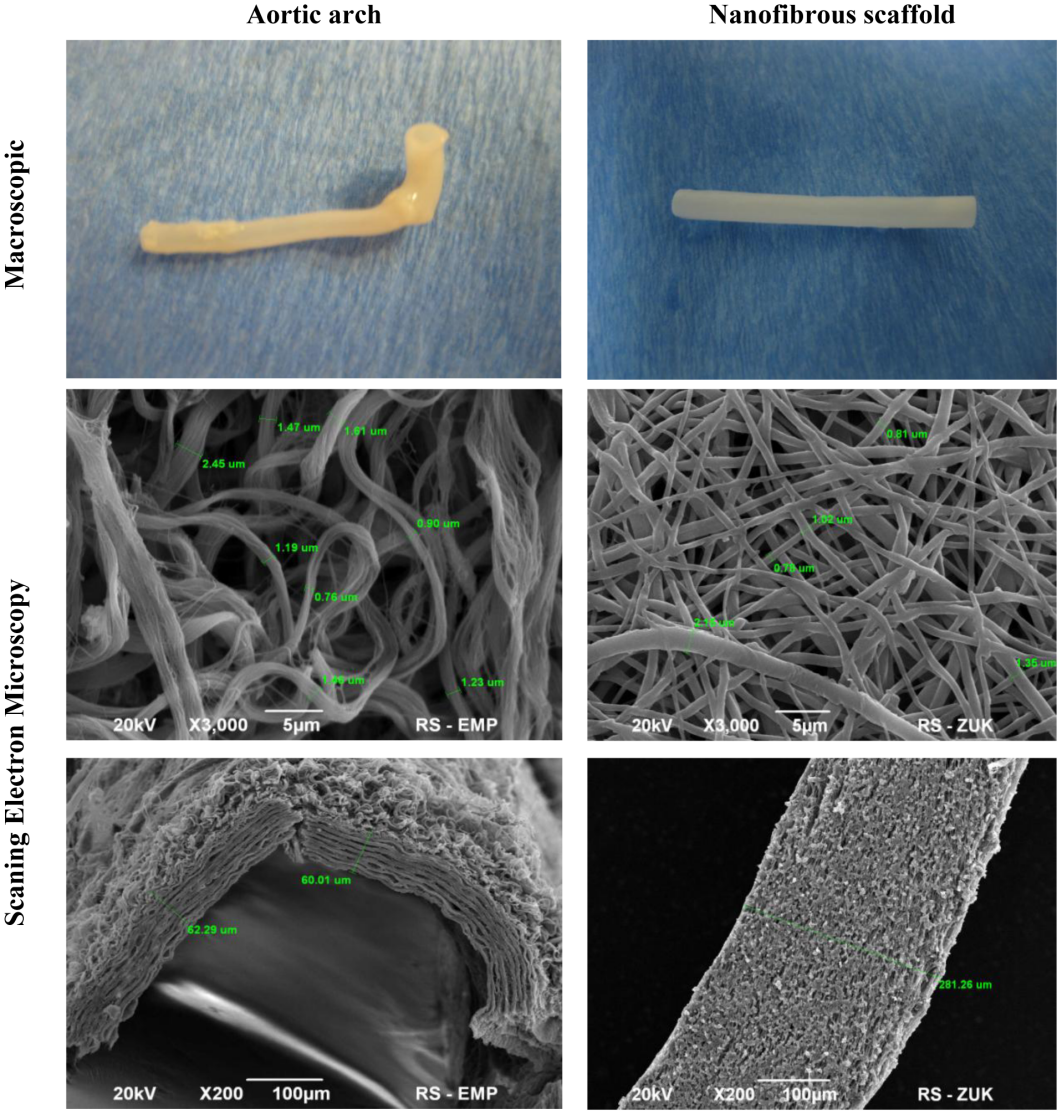
Macroscopic and scanning electron microscopy images of aortic arch and electrospun nanofibrous scaffolds.

No remnants of cell debris were detected throughout the cross-section of the aortic arches after decellularization (Fig. 1). Scanning electron microscopy showed high porosity of decellularized aortic arch scaffold built of submicron size filaments bundled to form fiber of thickness ranged from 0.9 to 2.45 µm and wall thickness 60–100 µm; multilayered structure of the wall was also revealed. Electrospun nanofibrous scaffold had also high porosity structure (ca. 78%), with smaller pores and higher fiber density compared to aortic arch. Unlike in aortic arch, the fibers were not formed of bundles. The fibers thickness of PLCL scaffold are similar to that of aortic arch, and ranged from 0.81 to 2.15 µm. The wall thickness is about 280 µm (Fig. 1).

### PLCL degradation test

**Figure 2:**
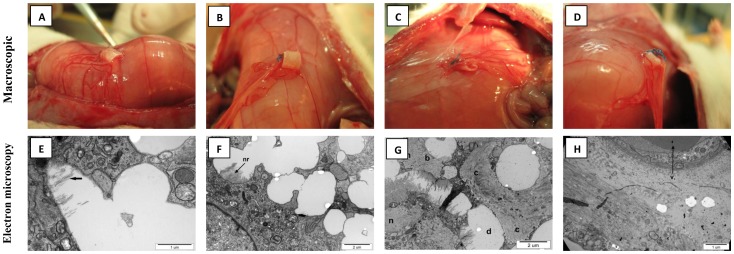
PLCL degradation test. A–D – Macroscopic evaluation, tested scaffold was covered with host tissue with well developed vascular network; E–H - Ultrastructural micrographs of specimen harvested from rat’s peritoneum 6 weeks after nanomaterial implantation. E - Arrow points bundles of collagen; F – (nr – nanomaterial residue); G – (b – border between collagen and nanomaterial, c – collagen, d – dissolved nanomaterial, n – niche filled with collagen); H - (v – blood vessel lumen).

Six week after PLCL implantation into rat peritoneum tested scaffolds covered with host tissue with well developed vascular network (Fig. 2A–D). Specimen analyzed by ultrastructural electron microscopy contained cells of proper shape. Absence of macrophages and lymphocytes indicated absence of inflammatory response. The majority of nanofibrous membrane was dissolved during the specimen fixation, but with different level of integration with tissue some residues are visible. Fig. 2E (**arrow**) shows delicate bundles of collagen fibrils replacing degrading electrospun membrane. Fig. 2F (**nr**-nanomaterial residue) shows the nanomaterial remains. The border between proliferating elements of connective tissue and nanofibrous material is distinctly shown on Fig. 2G (**b**-borders). Oval shaped cavities of different size are left by dissolved material are visible in the regions of less advanced tissue with material integration Fig. 2G (d). Collagen fibrils are filling regular, oval-shaped niches. (Fig. 2G **n**-niche). Proper blood vessels are formed in the nanomaterial (Fig. 2H **v** – blood vessel lumen). The specimens contained proper cells, no inflammatory response and pieces of nanomaterial on different stages of biodegradation (Fig. 2E–H).

### Cytotoxic assay

**Figure 3:**
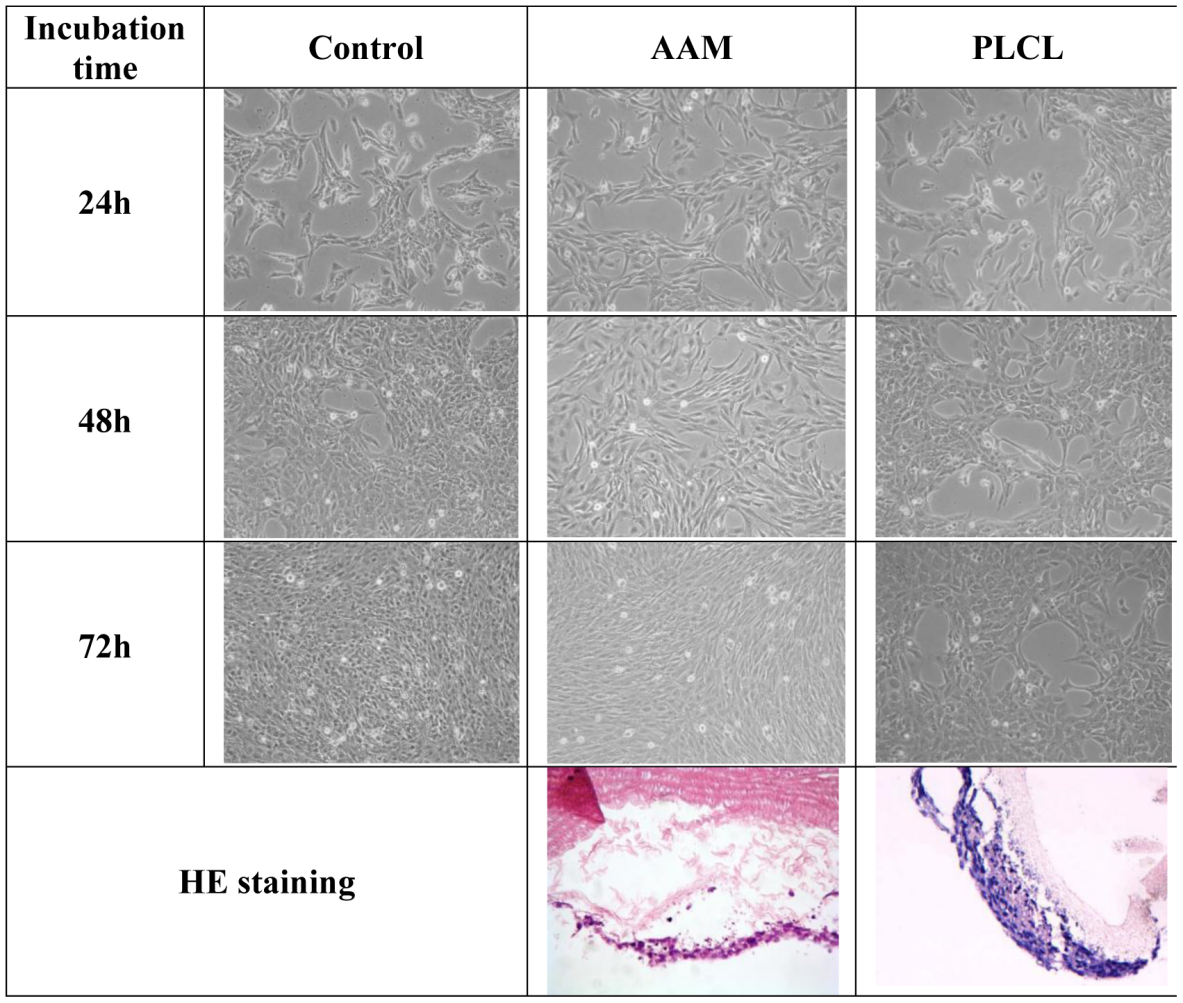
Cytotoxic effect of tested scaffolds on smooth muscle cell line. Cell morphology after treatment with conditioned media obtained after incubation with both scaffold type and hemotoxylin-eozin (HE) staining of cells seeded on tested scaffolds surface.

MTT assay showed no cytotoxic influence of both conditioned media obtained after 24 and 48 h incubation with acellular aortic arch and PLCL nanomaterial on CRL2018 cell line (Table 1). Slight cytotoxic influence was observed after 72 h incubation with conditioned medium obtained after incubation with PLCL scaffold. Microscopic analysis showed no differences in cell morphology after treatment with conditioned media compared to control (Fig. 3).

**Table 1:** Results of cytotoxic influence of conditioned media obtained after incubation with acellular aortic arch scaffold (AAM) and poly(L-lactide-*co*-caprolactone) (PLCL) on rat smooth muscle cell line (CRL2018).

Incubation time	Viability (%) compared to control	
	AAM	PLCL
24 h	102.1±15.2 (p = 0.76)	94.1±15.2 (p = 0.13)
48 h	92.8±19,8 (p = 0.40)	109.0±17.3 (p = 0.83)
72 h	98.8+12.0 (p = 0.83)	91.0±6.5 (p = 0.002)

### Cell growth analysis

After 2 weeks of cell culture HE staining showed growth of cell layers on both outer surfaces of tested scaffolds. Cells growth as a monolayer in case of acellular aortic arch scaffold. In case of PLCL scaffold cells created from one to several number of layers and started to penetrate inside the scaffold (Fig. 3).

### Surgical procedures

#### Urinary conduit construction

**Figure 4:**
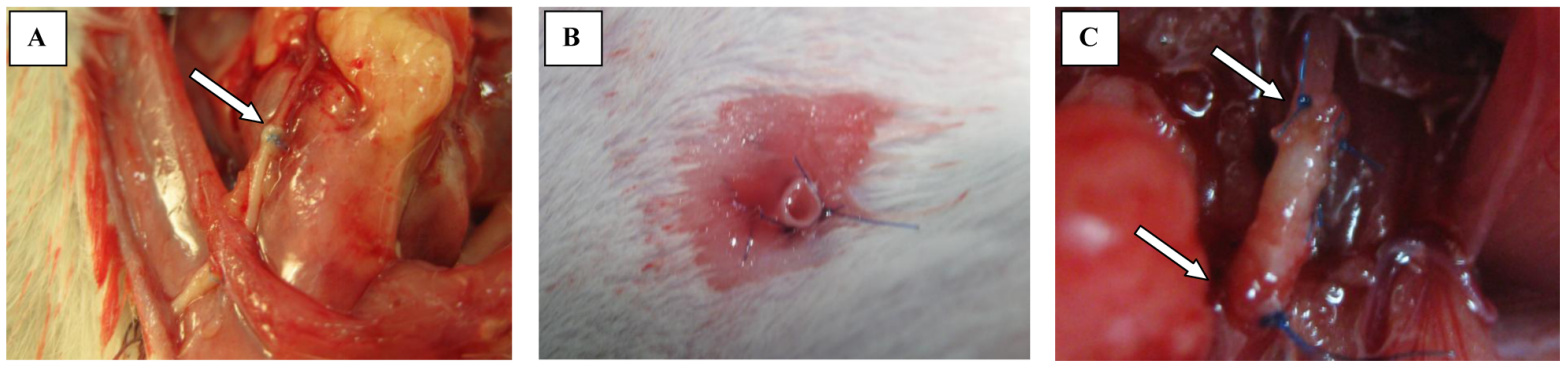
Effects of surgical procedure. A – End of urinary conduit reconstruction procedure, arrow marked site of end-to-end anastomosis; B – Stoma, urine leakage form constructed urinary conduit directly after end of surgical procedure; C – End of ureter segment construction procedure, arrow marked sites of end-to-end anastomosis.

All animals survived the surgical procedure. After the end of surgery urine leak from the conduit was observed in all cases at the outside at skin level (Fig. 4). Two rats in Group 1A and five rats in Group 1B completed the follow-up period (Table 2). There were no adhesions after the end of follow-up in all cases.

**Table 2:** Results of urinary conduit construction using acellular aortic arch and electrospun nanofibrous scaffolds.

No.	Time (day)	End of experiment	CE	Macroscopic evaluation			
				Upper urinary tract	Conduit lumen	CSS	Kidneys*
Group 1A – acellular aortic arch (AAM)							
1	8	Stoma atresion	Lack	Normal	Patent	Obstruction	120
2	8	Stoma atresion	Lack	Normal	Patent	Obstruction	109
3	5	Death	–	Normal	Patent	Patent	105
4	28	End of follow-up	Lack	Extended	Patent	Obstruction	138
5	28	End of follow-up	Lack	Normal	Patent	Obstruction	146
6	5	Death	–	Normal	Patent	Obstruction	110
Group 1B – poly(L-lactide-*co*-caprolactone) - PLCL							
7	28	End of follow-up	Lack	Extended	Patent	Obstruction	128
8	28	End of follow-up	Lack	Extended	Patent	Obstruction	132
9	14	Stoma atresion	Lack	Extended	Patent	Obstruction	116
**10**	**28**	**End of follow-up**	**Present**	**Extended**	**Patent**	**Patent**	**112**
11	28	End of follow-up	Lack	Extended	Patent	Patent	111
12	28	End of follow-up	Lack	Extended	Patent	Patent	124

Good functional results relating to all mentioned aspects (conduit, ureter, kidney) were bold. (CSS – conduit to skin anastomosis, CE – contrast excretion, Kidneys* - changes in kidneys size (in percentage) on the operated side compared to normal kidneys).

**Figure 5:**
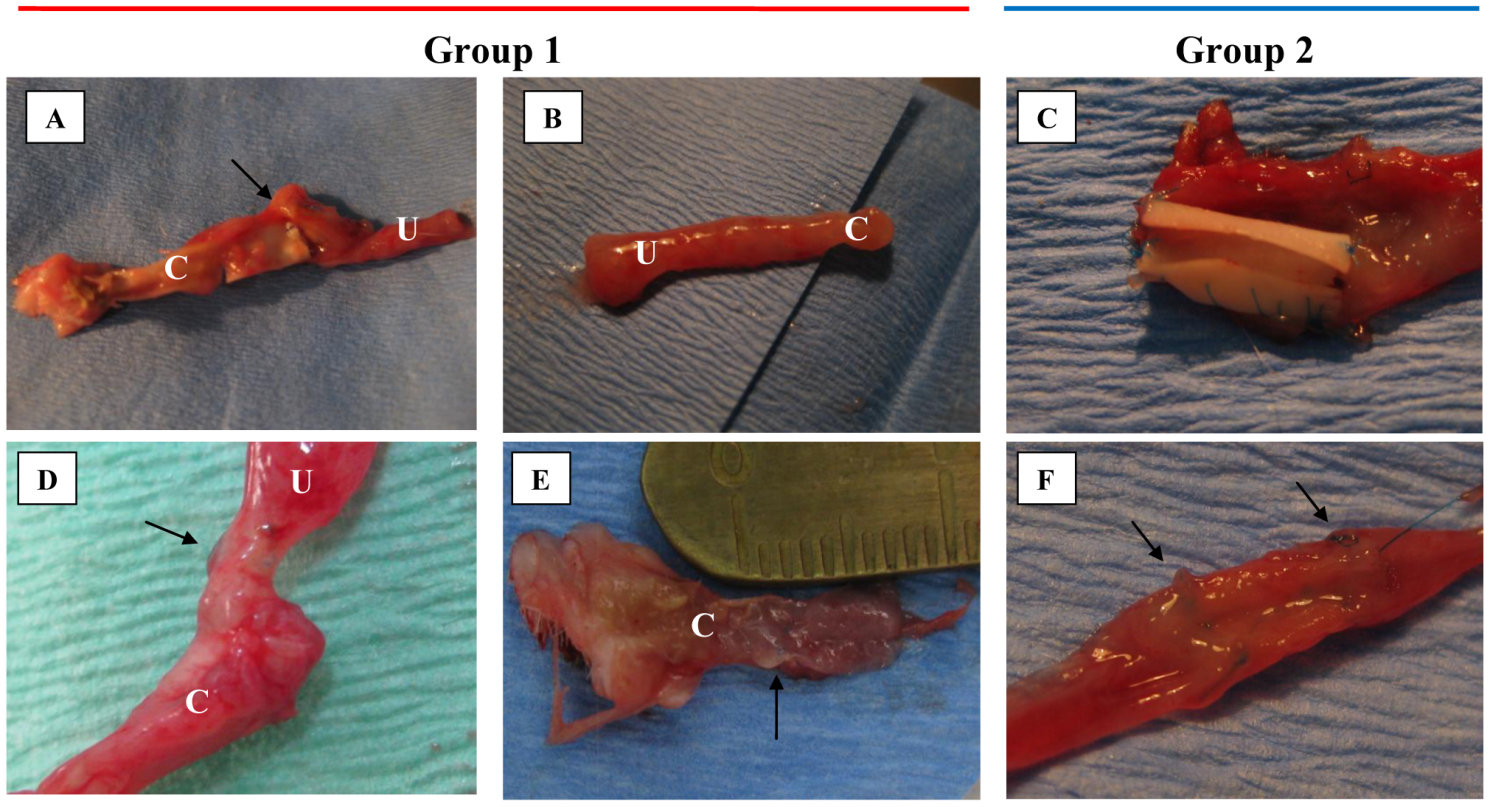
Integration of scaffolds with native ureters – macroscopic evaluation. A, C – lack of integration of electrospun nanofibrous scaffold; B – good integration of electrospun nanofibrous scaffold, it is impossible to find anastomosis site; D-F – good integration of aortic arch scaffold. C – conduit, U – ureter. Arrows marked sites of anastomosis.

**Figure 6:**
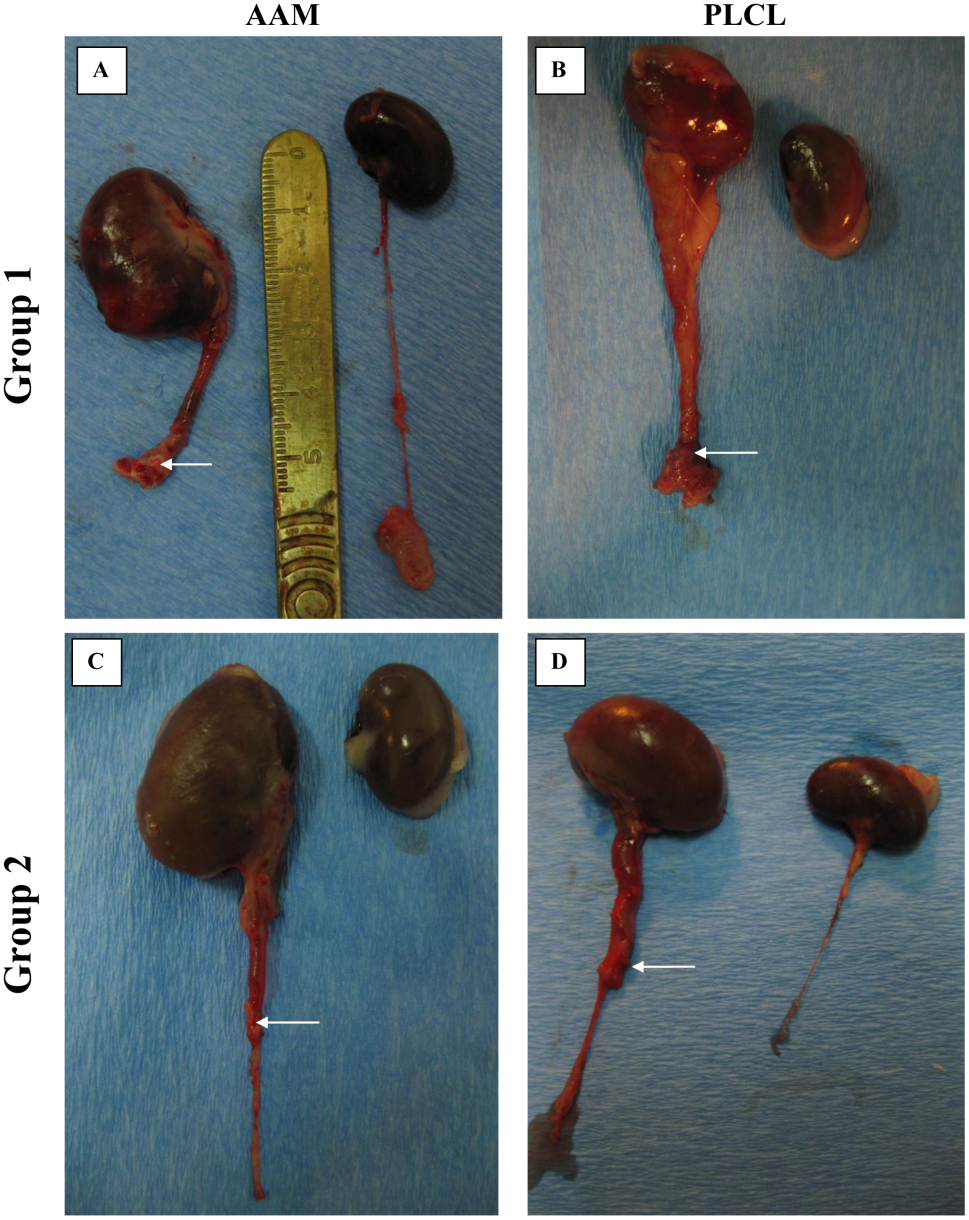
Macroscopic analysis of unsuccessful ureter regeneration in both tested groups. Kidney hydronephrosis and ureter extension can be observed. AAM – acellular aortic arch scaffold, PLCL - poly(L-lactide-*co*-caprolactone). Reconstructed segments are marked with arrows.

**Figure 7:**
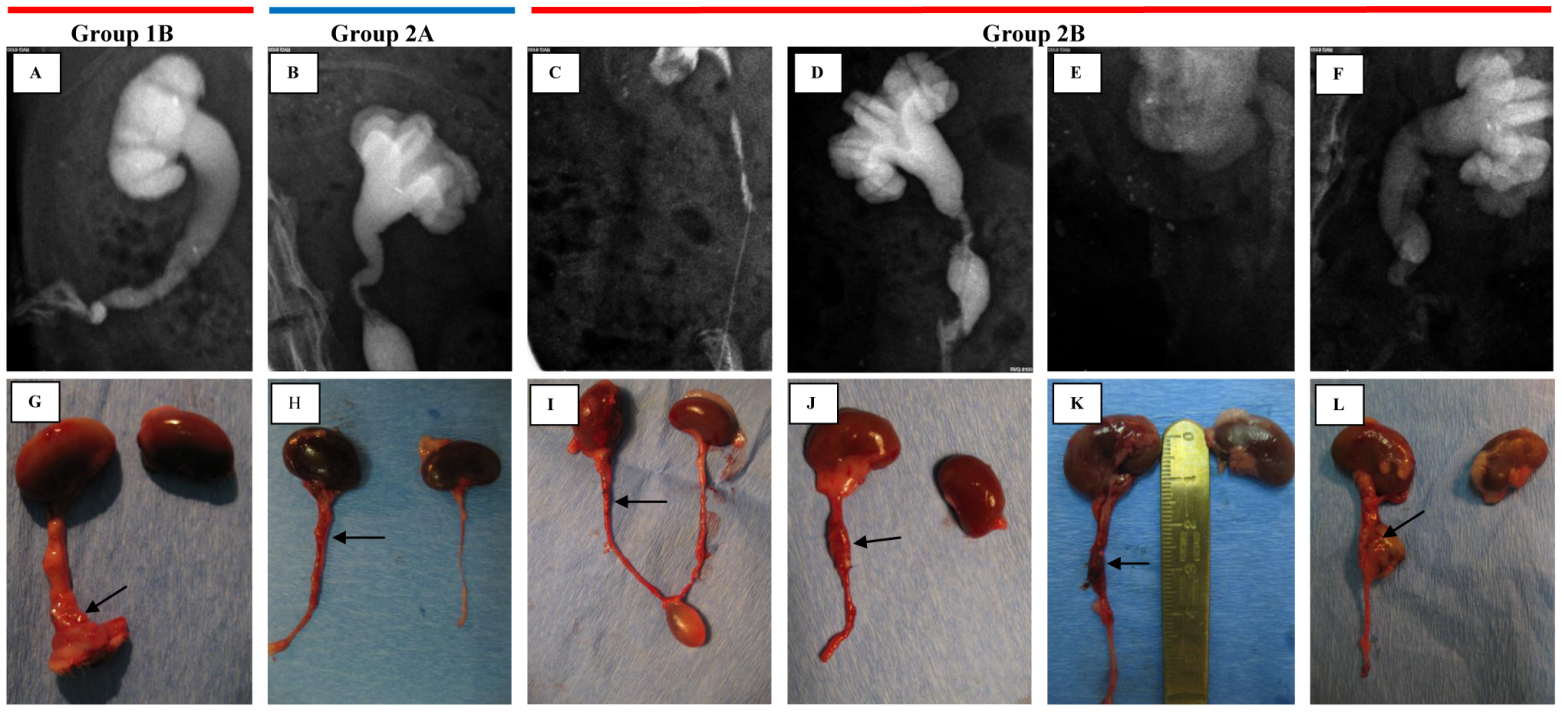
Successful ureter regeneration in all tested groups. A–F – urographic analysis, G–L – macroscopic analysis. A, G – urinary conduit construction using PLCL; B, H – ureter segment reconstruction using AAM, C–F, I–L - ureter segment reconstruction using PLCL. AAM – acellular aortic arch scaffold, PLCL - poly(L-lactide-*co*-caprolactone). Reconstructed segments are marked with arrows.

Aortic arch scaffold integrated better with native ureter than nanofibrous scaffold in gross examination (Fig. 5D,E). However the use of natural scaffold resulted in stenosis, obstruction and urine flow inhibition within one week (3–8 days) after surgery. At the end of follow up hydronephrosis and ureter extension were observed (Fig. 6A). Nanofibrous scaffold, showed worse integration with ureter than natural scaffold (Fig. 5A). For one case (rat no. 10) conduit resembled native ureter 28 days after surgery (Fig. 5B). Patency of the conduits was observed in 3 cases from Group 1B, but only in one case clear urine flow from conduit was noticed (Table 2). In this case intravenous pyelography showed a patent uretero-PLCL-conduit junction with mild hydronephrosis and mild ureter extension compared to unsuccessful cases (Fig. 6B, Fig. 7A,G).

#### Ureter segment reconstruction

All animals survived the surgical procedure. Four rats in Group 2A and six rats in Group 2B completed the follow-up period (Table 3). In one case in Group 2A and in four cases in Group 2B urographic analysis showed patent uretero-scaffold anastomosis (Table 3, Fig. 7B–F).

**Table 3:** Results of ureter segment reconstruction using acellular aortic arch and electrospun nanofibrous scaffolds.

No.	Time (day)	End of experiment	CE	Macroscopic evaluation		
				Upper urinary tract	Reconstructed ureter segment	Kidney*
Group 2A – acellular aortic arch (AAM)						
13	1	Death	–	–	–	–
14	28	End of follow-up	Lack	Extended	Patent	125
15	12	Death	–	Extended	Patent	116
**16**	**28**	**End of follow-up**	**Present**	**Extended**	**Patent**	**106**
17	28	End of follow-up	Lack	Extended	Patent	150
18	28	End of follow-up	Lack	Extended	Patent	141
Group 2B – poly(L-lactide-*co*-caprolactone) - PLCL						
19	28	End of follow-up	Lack	Extended	Patent	128
20	28	End of follow-up	Lack	Extended	Patent	139
**21**	**28**	**End of follow-up**	**Present**	**Extended**	**Patent**	**119**
**22**	**28**	**End of follow-up**	**Present**	**Normal**	**Patent**	**123**
**23**	**28**	**End of follow-up**	**Present**	**Extended**	**Patent**	**121**
**24**	**28**	**End of follow-up**	**Present**	**Extended**	**Patent**	**118**

Good functional results relating to all mentioned aspects (reconstructed segment, ureter, kidney) were bold. (CE – contrast excretion, Kidneys* - changes in kidneys size (in percentage) on the operated side compared to normal kidneys).

Macroscopic analysis showed hydronephrosis and ureter extension in rats in which intravenous pielography showed lack of visible urinary tract on X-ray images in two tested groups (2A and 2B, Fig. 6C, D). In other cases, in which urographic analysis showed patent uretero-scaffold anastomosis this side-effects were less severe. (Fig. 7H–L). Acellular aortic arch scaffold integrate very well with native ureter resembling its structure (Fig. 5F). Integration of synthetic PLCL scaffold with native ureter was not observed, similarly to lack of degradation of PLCL scaffold (Fig. 5C). Overgrowth of connective tissue over the synthetic scaffold was observed (Fig. 7L).

Both tested scaffolds have reduced their length by approximately 30% at the end of follow up. Catheters were find under reconstructed segment, in the lower ureter part near the bladder.

### Histological analysis

**Figure 8:**
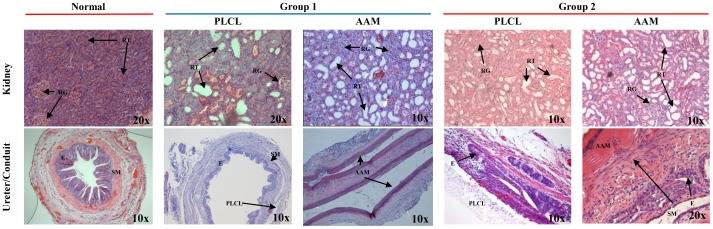
Histological changes in kidneys and tissue layer regeneration on ureter segments in both tested groups. RG-renal glomeruli, RT-renal tubules, E-epithelial layer, SM-smooth muscle layer. AAM – acellular aortic arch scaffold, PLCL - poly(L-lactide-*co*-caprolactone). Magnification was placed in right corner of images.

In Group 1 histological analysis of kidneys usually showed intense purulent inflammatory infiltration with renal tubule atrophy 28 days after surgery. In rat no. 10 this processes was less severe. Only slight degenerative changes in renal tubule and small focal inflammatory infiltration were observed. Less than 50% of renal tubules were enlarged (Fig. 8). In Group 2 inflammatory process with hydronephrosis was also observed. Renal tubules were enlarged which was not correlated with scaffold type and surgical procedure effectiveness (Fig. 8).

In Group 1, histological evaluation of aortic arch conduit showed intense inflammatory infiltration with lack of epithelial and smooth muscle layer regeneration. Tubular nanofibrous PLCL scaffold showed better properties as a conduit. Visible multilayered epithelium covering the PLCL conduit lumen and focal developed smooth muscle layer were observed (Fig. 8). In Group 2 on both scaffolds regeneration of urothelium and smooth muscle layer was observed. Inflammatory infiltration with fibrosis was also observed (Fig. 8).

**Figure 9:**
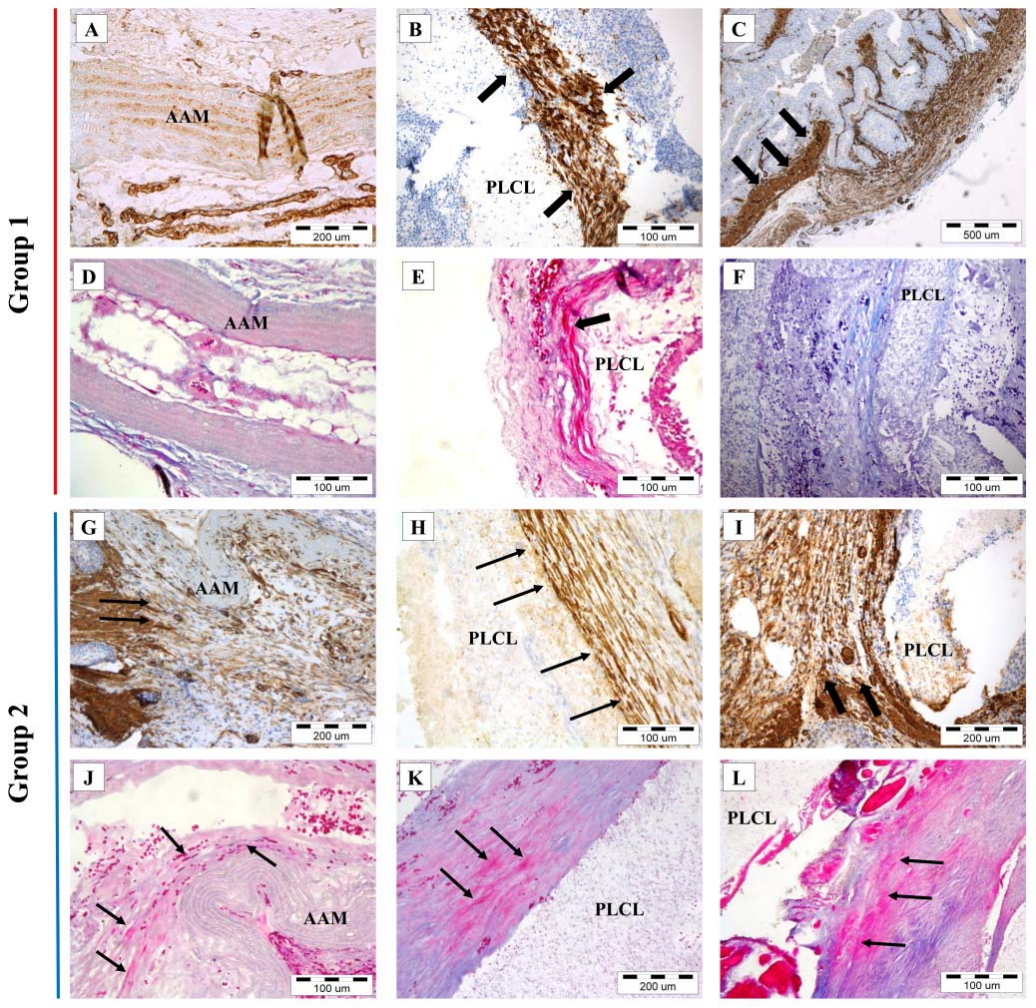
Confirmation of smooth muscle regeneration on both tested scaffolds by immunohistochemical (A–C, G–I) and Masson (D–F, J–L) analysis. A, D, F – Lack of smooth muscle layer regeneration. B, C, H, J–L – smooth muscle layer regeneration on both scaffold type and all tested groups. C, G, I – migration of smooth muscle cells form native ureter (arrows) to AAM and PLCL scaffolds. (Light microscopy). AAM – acellular aortic arch scaffold, PLCL - poly(L-lactide-*co*-caprolactone).

Regeneration of smooth muscle layer was confirmed by immunohistochemical and Masson staining. In Group 1A regeneration was not observed (Fig. 9A,D). In Group 1B intensive regeneration of smooth muscle layer with high cell density was observed. Creation of muscle bundles, migration of cells form native ureter, cell migration inside the scaffold and round cells in early growth phase were also observed (Fig. 9B,C,E,F). In Group 2 on both scaffold types regeneration was observed. Cell migration form native ureter on scaffold surface, cell growth in monolayer, chaotic layers of muscle cells and muscle bundles creation was observed (Fig. 9G–L).

### Statistical analysis

Fisher’s exact test have not detected dependency between scaffold type and reconstruction result both in Group 1 and 2 (p = 0.09 and p = 0.12, respectively). In Group 1 important results were obtained from two fraction analysis (p = 0.045), in Group 2 results were not significantly important (p = 0.06). Both statistical analysis of scaffolds alone, without grouping, showed significantly important results (p = 0.01) between scaffold type and reconstruction result favoring PLCL scaffold.

## Discussion

In the present study we compared two different materials for urinary conduit construction and ureter segment reconstruction: natural rat acellular aortic arch and tubular nanofibrous scaffold made of biodegradable synthetic polyester PLCL.

There are two main material factors that differ acellular aortic arch from nanofibrous material. High porosity and wettability of the biomaterial makes cell colonization and infiltration very facile, but also enables infiltration of urine through the scaffold that causes tissue irritation and is harmful to the cells. Low porosity and wettability of the nanofibrous scaffold makes cell colonization much harder (or even disables stable attachment of tissue to the nanofibrous polyester scaffold), but also creates the barrier that is impermeable to urine and isolates newly formed tissue from its harmful influence. Thin layer of decellularized aortic arch enables fast integration with the surrounding tissue, but also increases the urine infiltration with its harmful consequences. Thick layer of the biomaterial has very good insulating properties against urine infiltration, yet thick layer of material is far harder to be digested by cells and as a consequence can trigger tissue inflammation caused by long time contact with a foreign body. The porosity, wettability and thickness of the scaffold needs to be balanced for the best performance [28].

Six week after implantation to peritoneum PLCL scaffold was surrounded by developed omentum vascular network (Fig. 2A–D). Analysis using ultrastructural electron microscopy suggest gradual integration of tested membrane with rat tissue. The scaffold niches, filled with Extracellular Collagen Matrix (ECM), are likely to be the traces of degraded nanomaterial. Absence of cells of improper structure or irregular pieces of membrane suggests good integration of electrospun material with tissue. Bridges of collagen fibrils are connecting regions of ECM. They are growing through nanomaterial showing its gradual integration with tissue. Fibroblasts surrounding the nanomaterial are producing collagen. The collagen replaces biodegraded nanomaterial. This properties suggests proper integration of tissue with electrospun nanomaterial (Fig. 2E–H). According to Morita et all. [29] solid PLCL completely degrades in *vitro* (in saline in 37°C) during one year (comparing to 3–5 years for poly(L-lactide) and more than 5 years for poly(caprolactone). *In vivo* degradation of thin PLCL spongy implants was assessed by Jeong et all. [30]. They have found that custom made PLCL (50% L-lactide, 50% ε-caprolactone) implanted subcutaneously and seeded with smooth muscle cells had lost 19% of the original mass after 15 weeks. Number average molecular mass have decreased to 23% of initial value and the cells ingrown to the scaffold. The degradation occurred through random scission of the polymer chain. Authors also concluded that amorphic domains of PLCL have degraded faster than crystalline domains of PLLA. Contemporary work of Thapsukhon et all. [31] dealt with a model very similar to our works. Microtubes made of custom made PLCL was electrospun to produce microtubes for *in vitro* degradation assessment. Microtubes made of PLCL (67% L-lactide, 33% ε-caprolactone – composition very close to commercial material used in our study) have lost 29,8% of mass during *in vitro* 36 weeks degradation in saline at 37°C. According to Bandyopadhyay et all. [32] sponges made of 70/30 l-lactide/ε-caprolactone copolymer (PLCL) seeded with myoblasts undergone complete *in vivo* biodegradation 9 months after implantation.

In our study we used scaffolds alone, without seeded cells. Nanofibrous scaffold showed better properties for urinary conduit construction than acellular aortic arch scaffold but its integration with native ureter was generally worse. PLCL was made of hydrophobic polymer and was not specially treated to increase hydrophilicity. Therefore it showed worse integration with ureter than natural scaffold. Acellular aortic arch scaffold because of its natural origin indicates very good integration with native ureter but other properties, especially scaffold diameter, caused conduit occlusion at the end-to-side anastomosis. Aortic arch have the largest diameter in rat, that is why we choose this vascular graft for experiment. Both scaffold types used in this experiment were disinfected before implantation using PBS with antibiotic, as previously described [33]. Conditioned media obtained from both tested scaffolds were nontoxic after 24 and 48 h incubation with smooth muscle cell line. Slight cytotoxic effect was observed only after 72 h using PLCL conditioned medium, but cell growth analysis together with PLCL degradation test showed that cells growth well on PLCL surface starting to penetrate inside the scaffold (Fig. 2, Fig. 3).

We used nonabsorbable sutures which served as a markers in this experiment. Despite use of such sutures and short follow-up, lack of stone formation inside the ureters and bladders were observed. Other important aspect was stoma formation in Group 1. Flat stoma can be result of improper urine collection in the bag and possible problem with the bag sticking to skin. To prevent the urine leakage we performed a stoma in the form of “chimney” (part of scaffold protruded outside the skin) because the diameter was too small to form nipple stoma in rat.

Until 2012 only one paper about urinary conduit construction using tissue engineering methods was available [33]. In this work Drewa used SIS seeded and unseeded with 3T3 fibroblast cell line for urinary conduit creation. In three cases conduits were patent, seeding with 3T3 cells did not improve the results obtained, in this group inflammation process was more severe than in group with unseeded scaffold. In recent years another group described their attempts to make artificial urinary conduit using tissue engineering. Geutjes et al. [34] used scaffold built from collagen type I and VyproII synthetic mesh for urinary conduit construction in 10 female pigs (4 unseeded scaffolds and 6 seeded with urothelial cells), but no differences between seeded and unseeded scaffolds were observed. Another study was performed on 30 rabbits using bladder acellular matrix (BAM) seeded or unseeded (control) with urothelial cells. In this study bladder was removed and two ureters were sutured to constructed conduit [35]. All 24 rabbits with cell seeded BAM survived follow-up. Multilayered epithelium covering the conduit lumen and lack of severe complications were noticed. In control group (n = 6) 4 rabbits died until 1 month after surgery, in two other cases fistula has appeared. Lack of epithelial layer regeneration was observed. Similar study was presented recently by the same group [36].

One of the first attempt of ureter regeneration was performed on dogs using human or monkey umbilical cord treated with cyclophosphamide to remove morphological blood elements (leukocytes). Only in one case good results in long follow-up period (3 years) were obtained, in other cases different degree of renal failure was observed [37]. Other acellular natural scaffolds like SIS, decellularized ureter, scaffolds derived from vessels or synthetic materials like Gore-Tex (polytetrafluoroethylene) were used [38]–[44]. All this experiments failed because of complications or not significantly important segment reconstruction (0.3 mm).

The important aspect in ureter regeneration is to maintain ureter continuity. Small tissue section in reconstructed segment can stimulate cell layers regeneration on scaffold. Additionally peristaltic wave is not interrupted. Promising results obtained after using onlay technique shows how difficult is regeneration of whole-diameter ureter segment [45]–[49].

Use of appropriate scaffold can stimulate its remodeling. In our study urothelium regeneration was observed on both tested scaffolds. Regeneration of smooth muscle was confirmed by immunohistochemical and Masson staining. The same effect was observed when SIS was used. Liao et al. showed lack of epithelium regeneration on unseeded BAM [35], [36]. Our experiment with BAM used for bladder regeneration showed urothelium regeneration on unseeded scaffold, but it has to be emphasized that urothelial layer growth is not an urinary tract regeneration [8]. Moreover, numerous clinical trials concerning bladder reconstruction proved that urothelium self-regenerates completely, migrating from ureters even after radical cystectomy [50], [51]. In our opinion scaffold preseeding only with urothelial cells is not necessary, especially for small scaffold such as used for urinary conduit creation because urothelium can easy self-regenerate from surrounding tissue. We think, that the important fact is provide scaffold protection from urine leakage [28].

## Conclusions

This experiment showed that construction of tissue engineered urinary conduit and ureter segment regeneration is possible. Electrospun nanofibrous scaffolds made of PLCL copolymer showed better properties than naturally derived aortic arch. Limitation of this study was small number of animals in each tested group, small animal model and short follow-up. On the other side rat is suitable animal model for experimental study for scaffolds properties.
